# Simulation in Regional Anaesthesia: A Narrative Review of Its History, Evolution and Future Prospects

**DOI:** 10.3390/jcm14010067

**Published:** 2024-12-26

**Authors:** Ashish Ranjan Satapathy, Iskandar Bin Khalid, Shahridan Mohd Fathil

**Affiliations:** 1 Department of Anaesthesia, Ng Teng Fong General Hospital, Singapore 609606, Singapore; satapathyashish@gmail.com; 2 Department of Anaesthesiology and Intensive Care, Hospital Canselor Tuanku Muhriz, Universiti Kebangsaan Malaysia, Kuala Lumpur 56000, Malaysia; 3 Department of Anaesthesiology, Gleneagles Hospital Johor, Iskandar Puteri 79250, Malaysia

**Keywords:** regional anaesthesia, simulation, simulation in regional anaesthesia, AI and artificial intelligence

## Abstract

Regional anaesthesia has seen a resurgence of sorts since the widespread advent of ultrasound into clinical practice. The ability to access hitherto inaccessible nerves and fascial planes in the human body whilst ensuring visualisation of the needle tip during block performance has opened the proverbial floodgates leading to its widespread adoption, further supported by a growing body of evidence for its many benefits in a patient’s perioperative journey and pain management. The concomitant advancement of technology and the development of powerful simulation and artificial intelligence tools has given a much-needed impetus towards improving training and safe practice in regional anaesthesia. Methods: We performed a detailed search of databases, including PubMed Medline, Web of Science, EBSCO, Embase and the Cochrane Library, up to October 2024. Our search was conducted using phrases including (but not limited to) “history of anaesthesia”, “history of simulation”, “regional anaesthesia and simulation”, AI and “artificial intelligence and anaesthesia”.

## 1. Introduction

Regional anaesthesia (RA) has long been the cornerstone of anaesthesia practice. RA, when used alone or in combination with GA, has been shown to provide improved perioperative analgesia [1,2,3,4], decreased post-operative complications [5,6,7,8], increased patient satisfaction [1,9] and an additional benefit of reduced cost [10,11]. In spite of these glaring advantages, RA, until fairly recently, has been an art practised by a dedicated and privileged few [12,13]. Limitations of ultrasound technology resulting in the inability to visualise the target nerve, the approaching needle and vital structures in the vicinity of the target had put this now invaluable asset on the backburner for decades.

This pendulum shifted in 1994 when Steven Kapral revealed the sonoanatomy of the brachial plexus using B mode ultrasound [14], inadvertently unleashing this powerful tool for the benefit of all anaesthesiologists and catapulting ultrasound-guided regional anaesthesia (UGRA) to the forefront of anaesthesia practice. Once the floodgates opened, there was no dearth of newer generations of ultrasound machines featuring superior resolution, features and portability, improved echogenic needles and enthusiastic clinicians who set out to stamp their authority in this emerging field [15].

Nevertheless, despite the widespread use of ultrasound in RA, the incidence of RA-related complications did not drastically decline as expected [16]. Conversely, there was an initial increase in case reports of unexpected complications [17,18,19], likely a consequence of the increasing number of blocks performed and the over-enthusiastic adoption and application of ultrasound for more challenging blocks.

Clinicians soon realised that attaining proficiency in UGRA requires a steep learning curve in comparison to traditional nerve-stimulation and landmark-based techniques due to the greater number of variables affecting successful block performance. In addition to sound anatomical knowledge, clinicians now needed to acquire an additional understanding of the sonoanatomy of each block, principles of practical ultrasonography, as well as the ability to mentally depict three-dimensional images from the two-dimensional picture seen on screen, recognize artefacts, and exclude background noise [20,21,22,23]. Furthermore, honing motor skills was required to improve hand-eye coordination, an essential skill for in-plane and out-of-plane needle alignment with the ultrasound probe whilst simultaneously maintaining focus on the target structure in the ultrasound image [24]. With ever-changing technology, the tools and techniques used in UGRA training have evolved to meet this required level of coordination between sensory and motor skills. Over the last decade or so, UGRA training has grown to incorporate high-fidelity simulation to ensure the next generation of RA practitioners are able to effectively use the powerful tools now at their disposal.

## 2. Why Do We Need Simulation in Healthcare

A 2013 patient-safety report from the United States revealed some alarming statistics: preventable medical errors have resulted in more than 400,000 yearly deaths, making it the third leading cause of mortality in the country [25]. Moreover, iatrogenic medical errors have resulted in 3.5 million per year experiencing permanent harm and disability in the United States [25]. The authors from the “Journal of Patient Safety” even went on to opine that:

“*One of the main reasons for such alarming statistics may be related to the medical education culture. Since the Flexner report [26], many advances have been made in technology and teaching strategies; however, it is still not unusual for medical students to be taught almost the same way they were decades ago. Evidence-based methodology, patient safety, andragogy, accessible, high-quality media production, computers, smartphones, the Internet, 3D printers, high and low-fidelity mannequins—most of this is basically not taken into consideration when defining the curriculum and the pedagogical methods to shape and enhance the background of future health care professionals*”.

The aerospace industry, where there is little margin for error akin to the healthcare industry, has quickly embraced many of the advances in simulation-based learning models, leading to a shift in safety rating of “risky” towards the end of the 1950s to “safer” in short period of several years [27]. Aviation simulation dates back to 1929 when Edwin Albert Link was the first to invent a flight simulator, a prototype model he coined the “Blue Box”. This novel simulator was a fuselage-like device consisting of a cockpit and a control interface [28], which was later marketed as the “Pilot Maker”. The simulator’s ability to replicate flying motions and sensations enabled Link to train his own brother to pilot an aircraft within the same year. The year 1934 saw a series of American postal carriers’ crashes attributed to pilot error in “poor meteorological conditions”, which continued despite the contracting of the US Army Air Corps [29]. It was during this time that attention turned to the Link Pilot Maker, which, once utilised, produced results which were astounding, to say the least; pilot preparedness in the face of adverse weather conditions was significantly improved, and the number of accidents decreased dramatically. As a result, the simulator quickly became a mandatory component of pilot training in numerous countries [30,31]. The success of the Blue Box/Pilot Maker provided concrete evidence that simulation-based training (SBT) could be successfully applied in many human endeavours. It became apparent that simulated flight provided a controlled and protected environment where trainee pilots could be subjected to simulated high-risk scenarios that would not have been possible without endangering invaluable lives. In addition, the training was standardized, reproducible and could mimic real-world conditions with increasing levels of complexity, which allowed pilots with varying competence levels to attain flight proficiency and preparedness for adverse weather conditions. Repeated exposure to such situations in a simulated environment made the “learning objective” part of their subconscious, and they were able to spontaneously maintain a similar level of performance in live crisis situations. While the healthcare industry was slow to adopt simulation training as part of the curriculum, the aerospace industry, despite its relatively young age, has swiftly set SBT as the mandated standard for the training of pilots and other aviators [32].

## 3. Application of Simulation in Healthcare

It had become apparent from the damning 2013 patient safety report [18] that current pedagogical methods of training had become obsolete. Even before the aforementioned report, Choy et al. had remarked that the traditional concept of didactic teaching and the “see one- do one- teach one” approach had proven to be inconsistent, ineffective, time-consuming and an expensive model for teaching complex procedures to surgical trainees [33]. This belief has since been found to be equally applicable to all branches of medicine where complex procedural skills are required, including anaesthesia and intensive care.

In a BMJ article published in 2008 titled “Teaching Procedural skills”, Grantcharav and Reznick aptly wrote [34]:

“*See one, do one is no longer appropriate for educating health professionals to perform complex procedures. Graduated independence, the hallmark of the approach to teaching procedural skills, is being challenged by concerns for patient safety, the skyrocketing complexity of procedures, and a diminishing work week for trainees. Finding the balance between patient safety and physician training will require a more structured approach to our skills curriculum, including continuous assessment of skills, constructive feedback, and provision of opportunities for deliberate practice in the teaching environment*.”

Simulation ensures delivery of this “teaching environment”, which is safe, controlled, repeatable and reproducible, where problem-based learning is practised, and competences are developed to a high standard, ensuring the patient is protected from the novice learner. The authors further recommended that three sets of essential skills should be mandatory components as part of “Pre-Patient Training” in any high-procedural-skill speciality and that they should be done outside the clinical setting to protect patients. These include the following:

- Gaining a cognitive understanding of the specific procedure, including its steps, the function and the operation of the associated equipment.
- Receiving training in fundamental, generic enabling skills necessary for the procedure.
- Opportunity to carry out the procedure across multiple platforms, including virtual reality, bench model simulators, cadavers and live animal models.

The use of SBT in UGRA addresses the 3rd aspect of the required skill set described above by facilitating target image acquisition and identification and improving needling techniques via improved hand-eye coordination.

## 4. Why Do We Need Simulation in Regional Anaesthesia

RA, with its inherent procedural complexities, has not been immune to the deficiencies in teaching and training described above. The ASA Closed Claims Project, which serves as a reporting system that offers an indirect evaluation of anaesthesia practice safety in the United States, in its 1999 report, found that though the incidence of death and brain damage had started to decline from the 70s to the 90s, the incidence of non-fatal nerve injury had actually increased from 15 to 18% [35]. Though the Project represented a national quality-assurance system, albeit without a denominator, the closed claims data uncovered significant and previously overlooked aspects of adverse anaesthetic outcomes, including nerve injury. Focused teaching and training in airway techniques had led to a reduction in airway-related critical incidents and patient harm in the decades prior to the report, but the same outcome was not seen in the field of RA. This again highlighted the issue of lack of structured training in RA and the importance of practising blocks in simulated environments to reduce patient harm. The parallel advent of ultrasound at this time had necessitated the acquisition of a new set of skills with a steep learning curve, and educators soon realised that this had to be imparted at an early stage for anaesthesia trainees to have a meaningful impact on the quality of RA training and reducing patient harm. Hence, began the arduous task of identifying areas of RA training that anaesthesia trainees were lacking and where simulation could make a difference.

One such study from 2006 involved six anaesthesia residents who performed 520 peripheral nerve blocks on live patients over a one-month period [36]. Five quality-compromising behaviours were identified: (a) inability to recognize local anaesthetic maldistribution, (b) inability to detect an intramuscularly sited needle tip, (c) fatigue, (d) inability to synchronize patient and image laterality and (e) inappropriate selection of needle insertion site and angle relative to the probe, leading to poor needle visualisation. The two commonest errors identified in the study were the inability to achieve needle visualisation prior to needle advancement and non-intentional movements of the ultrasound probe. Based on the analysis of the committed errors and identification of quality-compromising behaviours, the authors identified key learning objectives for future training and simulation programs.

In a more recent review covering 28 studies from 2009 to 2023, Ashokka and colleagues looked specifically at the “educational outcomes related to the implementation of simulation in RA” [37]. Though the simulation platforms were heterogeneous, they discovered that two studies actually achieved reduced incidence of paraesthesia and clinical complications. What was even more encouraging to see was that the improvements in Lab settings and Clinical settings were seen in 12 and 11 studies, respectively, which accounted for 82% of the studies included. Furthermore, knowledge improvements and self-reported improvement in confidence were seen in 2 and 1 study, respectively. They have inferred from the available evidence that with the use of hybrid simulation techniques, we should be able to achieve sustained improvements beyond 6 months.

## 5. Simulators in Regional Anaesthesia Training

Simulators for the purposes of RA training have traditionally been classified into two groups based on fidelity, defined as the degree of exactness with which something is copied or reproduced [38]:

1. Low Fidelity: also called Part-task trainers. Models vary from simple homemade gelatin or agar constructs to fresh frozen cadavers. A prospective observational study comparing the commercial Blue Phantom model [39] with homemade gelatin and tofu models showed that after the costs of each model were considered, participants preferred the gelatin model despite the seemingly greater fidelity of the Blue Phantom [40]. Recently, with the introduction of 3D printing technology, it has been possible to print custom-made parts of the human body on which specific and simple tasks can be practiced and taught to trainees. Such models are invaluable for attaining spatial orientation to three-dimensional structures such as the deeper nerves and neuraxial blocks [41]. Another low fidelity but invaluable model available in recent times are Thiel cadavers, a form of flexible, soft-embalmed cadavers which feature acoustic and hydro dissection properties similar to that in vivo, yet with the advantage of greater durability compared to fresh frozen cadavers [42]. Widespread use of Thiel cadavers has been limited by cost, and there is evidence to suggest that meat-based phantom models are as effective as fresh frozen cadavers for teaching ultrasound-guided needling to novice practitioners [43]. This suggests that cheaper, cost-effective models can be used effectively for part-task training as long as the training objectives are suitably matched.
2. High Fidelity: also called Complex-task trainers. High-fidelity simulators usually involve multiple tasks and are either mannikin-based, screen-based, role-play or a hybrid system incorporating features of all three. The mannikin-based simulators can be used to run simulation scenarios for unanticipated adverse events related to RA, such as local anaesthetic systemic toxicity (LAST), with the scope to incorporate advanced life support while providing ample opportunity for observation and feedback. Screen-based simulators are used to teach anatomy, sonoanatomy and three-dimensional spatial orientation of complex structures. Currently, there are several high-fidelity simulators available on the market that can simulate the performance of central neuraxial blocks, intermediate and deep peripheral nerve blocks and even interfascial plane blocks (e.g., BlockSim^®^). Torrano et al. studied the utility of SBT as part of a 4-h RA training workshop where first-year anaesthesia residents were given two attempts on a high-fidelity erector spinae plane (ESP) block simulator, first without previous practice and second at the end of the course [44]. Their proficiency in UGRA appeared to be immediately enhanced as their second attempt yielded significant improvements in time to block performance, number of needle insertions and ability to accurately aim at the ESP.

High-fidelity simulators utilizing immersive virtual environments such as augmented reality (AR) and virtual reality (VR) have also significantly increased in popularity in recent years [45]. While AR complements and enhances the real world by overlaying digital objects onto the user’s surroundings, VR immerses the user in an entirely separate digital environment, often with a head-mounted display unit and hand-held motion controllers, which allow interaction with the virtual environment. Proponents argue that VR-based simulators can facilitate RA training without geographic constraints and limitations, thus avoiding the financial, temporal and environmental costs of travel while presenting no safety risks to patients [46]. Additionally, data regarding the learner’s performance can be easily stored and analysed for assessment and feedback at a later date. Nevertheless, Chuan et al., in a recent randomised controlled trial, found that RA novices who were trained using a program based on a virtual reality simulator designed and validated by the same team were not superior to those trained using a conventional teaching program in terms of a global rating or composite error score [47].

The use of high and low fidelity does not capture the entire gamut of available simulators. To mirror this sentiment, a new method of classification has been proposed [48]:

1. Physical fidelity: the simulator looks and feels real (e.g., an intravenous cannulation hand model)
2. Functional fidelity: the simulator might look different but achieves the purpose (is functional) of part-task training (e.g., using a banana to simulate loss of resistance during epidural insertion)
3. Psychological fidelity: produces an effect on the user identical to the actual experience and includes role play and hybrid simulation models.

High fidelity in all aspects is not required for effective simulation training. However, it is crucial to align the level of fidelity with the intended learning objectives. An alternative to an expensive high-fidelity model would be a series of part-task training models designed to teach a particular task along the continuum. When simulation training is more expensive and resource-intensive than traditional didactic teaching, a curriculum blending both educational modalities may offer the most cost-effective strategy. Available evidence strongly suggests the importance of incorporating simulation in RA training in some form or another to achieve a certain level of competence before its application to live patients [49]. A meta-analysis of simulation vs. non-simulation training by Cook et al. showed improvement in all measurable learning outcomes, such as time taken, procedural flow, and successful task completion. In this study, simulation was found to be most effective in developing technical and professional skills as opposed to enhancing theoretical knowledge [50]. Review articles and a systematic review have also confirmed the usefulness of simulation when integrated into RA teaching [51,52,53] with the advancement of technology and incorporation of artificial intelligence (AI) into all aspects of life, including healthcare, we should aim to take advantage of the opportunities to develop powerful but cost-effective tools to train our next generation of RA practitioners. We agree with Chen et al., who argued that the RA fraternity should come together to a consensus and adopt standardized ‘core outcome sets’, which will act as a framework for RA simulation research and ensure greater standardization of studies for systematic reviews in the future [53].

## 6. Artificial Intelligence in Regional Anaesthesia

Artificial intelligence (AI) is an area of computer science focusing on techniques that enable computers to mimic human intelligence [54]. With the development of quantum computing and exponential growth in computational speed, AI has evolved from its initial “replication” stage into being an autonomous, self-learning and adapting tool. Due to the ability of computers to process big data and breeze through millions of algorithms at a fraction of a second in real-time, these machines can perform complex tasks, identify and warn about deviations from the norm, suggest corrective measures that can aid decision-making whilst remaining indefatigable and ready to be used for the next task at the click of a button. There lies ample opportunity for the use of AI in RA to assist in training, pattern recognition, needle-tracking, haptic feedback, visualisation of LA spread, recognition of injection pressures and overall decision-making. The use of AI in RA has been studied in the following areas:

1. **Needle tip guidance systems**: Hand-eye coordination and real-time needle tip visualisation remain one of the most difficult aspects of RA to teach and master [24]. In a study conducted on anaesthetists and novice medical students, McLeod and colleagues demonstrated that the use of a novel needle-tip tracking system led to improved needle tip visualisation, needle alignment to the ultrasound transducer and needle advancement, with 75% of subjects showing enhanced performance over time with tracking technology [55]. More recent studies have added further credence to novel needle guidance systems and their utility in training [56,57,58,59].
2. **Augmented reality systems**:
  - **Motion**: One of the most important aspects of RA training is gaining proficiency in the fine motor skills needed for safe needling. The Imperial College surgical assessment device (ICSAD), a validated system to analyze hand motion, has been widely used for objective assessment of technical skills during surgical training. The use of this tool in performing ultrasound-guided supraclavicular brachial plexus blocks showed notable differences in performance between RA experts and novices, including variations in time taken, number of movements, and path length travelled by each hand. Additionally, it demonstrated tangible improvements in the novices’ performances as they progressed through their RA fellowship [60]. A different group of investigators studying hand-motion analysis to assess needle tip tracking technology on a pork phantom noted a decrease in the number of hand movements and path length, but this was observed only for out-of-plane blocks [57]. A similar improvement in hand movements and path lengths was also seen in a study on volunteers performing a lumbar plexus block with needle tip tracking [58]. Although hand motion analysis does provide valuable information about the role of hand movements for specific tasks, as well as the economy of movements and efficiency, it does not provide a comparable evaluation of hand-eye coordination. Tools have been developed to evaluate hand-eye coordination in UGRA using self-assessment video-based methods [24], but without a metric for visual attention, these assessments remain somewhat subjective.
  - **Vision**: Correlating anatomy with sonoanatomy and forming a spatial orientation of identified structures is a requisite skill in UGRA that takes time to develop. Novice RA practitioners primarily depend on a selective visual processing pathway, utilizing limited top-down processing [61]. Their visual search is a slow process involving a sequential examination of one feature at a time that aligns with their explicit expectations. Moreover, this depends on the extent of background knowledge of the trainee, [62] hence the importance of didactic teaching methods in addition to simulation training. In contrast, experts can integrate top-down knowledge with holistic visual pattern recognition (i.e., bottom-up saliency), creating an implicit priority map that allows for quicker and more precise visual scanning [63]. This also allows them to direct more attention to task-relevant areas in accordance with the information reduction hypothesis [64]. Eye-tracking, which was first studied in laparoscopy, radiology and pathology [65], has now been applied to UGRA to assess decision-making and attention allocation objectively. This technology can help identify the difficulties encountered by individual trainees, thus allowing for more focused attention while also being used to cluster trainee performance levels and track their learning curve. Recent technological innovations in this field include neural network-linked automatic calibration of glasses and software that offers real-time performance updates, which can be monitored over multiple nerve blocks. UGRA studies utilizing eye-tracking technology [66,67,68] show that eye movements can differentiate experienced RA practitioners from novices. Additionally, reflective feedback based on real-time performance has the potential to expedite the UGRA learning process.
  - **Touch**: While the scanning phase of UGRA depends largely on visual attention, needling skills rely on haptic feedback. An example of a haptic simulator is the Simulator of Anaesthesia for Loco-Regional Procedures (SAILOR) system, which uses three-dimensional rendering on a desk-mounted virtual system controlled by a mouse and keyboard [69]. However, validation for this device was limited to self-reported satisfaction scores. The Regional Anaesthesia Simulator and Assistant (RASimAs) system is another tool that combines virtual feedback using MRI or CT images of real patients with haptic feedback through grounded haptics [70]. On a wider scale, grounded kinesthetic haptics have provided a more realistic feedback experience, although this has not always translated to improved performance in areas such as laparoscopy [71,72]. Further progress has been made with ungrounded cutaneous haptics, which uses vibration feedback, and this approach has been employed with the Intuitive Surgical Da Vinci Standard robot (Intuitive Surgical, Inc., Sunnyvale, CA, USA), showing some evidence of improvement in performance [73].
3. **Robotic technology:** the use of mechanical robots in anaesthesia is still in its early stages, primarily explored in tracheal intubation and RA. A notable application is the Robotic Endoscope Automated Laryngeal Imaging for Tracheal Intubation (REALITI), which provides real-time image recognition and automated orientation for intubation [74]. Initial tests on mannequins showed that untrained individuals performed better with REALITI’s automated mode than with manual control. Another example is the use of the DaVinci system for performing single-shot nerve blocks and inserting perineural catheters under ultrasound guidance on a phantom model [75]. The Magellan robotic system, designed for semi-automated UGRA, features an arm that holds a nerve block needle at its tip, controlled by a joystick and software system [76]. Nevertheless, excessive reliance on robotic assistance for RA training should be avoided as although it may reduce performance variability among trainees, it could ultimately undermine overall competence. Overreliance on such technology could present risks in emergencies or during equipment malfunction. Therefore, it is important to integrate robotic technology in RA as feedback tools that support and complement, rather than replace, the learning process.

Cognitive robots, also known as clinical decision support (CDS) systems, serve to complement the RA operator’s skills and can be valuable in both training and clinical application. These systems fall into two main categories: rule-based expert systems, which rely on algorithms developed by specialists in the field, and machine learning systems, which improve by identifying recurring patterns in data from patient procedures [77]. An example is the SAFer Injection for Regional Anaesthesia (SAFIRA), an innovative device that eliminates the need for an assistant during nerve blocks while allowing for syringe aspiration and cessation of injection if the pressure exceeds 15 psi [78]. As these systems continue to evolve, they hold the potential to enhance the practice and safety of RA.

## 7. Barriers to Implementation of AI in Regional Anaesthesia

It is apparent that AI has immense potential to influence the way we teach, assess and perform RA techniques. Nonetheless, it is important to recognise the limitations and barriers to its implementation in our day-to-day practice:

1. **Governance**: In addition to ensuring stability and transparency, AI governance is crucial to account for the rapid changes that technological innovation brings. This is akin to clinical research, where ethical considerations alleviate potential harm by providing values and principles that guide researchers. Governance procedures should be adopted for AI, similar to governance frameworks for clinical trials. The Alan Turing Institute provides guidance on artificial intelligence ethics and safety; its framework of ethical values is referred to as the ‘SUM values’ [79]. These embrace respectfulness, openness, inclusivity and justice. Because AI systems lack accountability, the institute has developed ‘FAST track principles’ based on fairness (data, design, implementation, outcome accountability, sustainability (safety, accuracy, reliability, security, and robustness), and transparency in order to gain public trust. AI governance may also ensure compliance with the regulatory bodies involved in healthcare, which ultimately need to be convinced of the utility of emerging high-fidelity simulation systems.
2. **Cost:** A significant barrier to the implementation of AI platforms in RA is the potential costs involved. While the costs can be justified over the longer term, in terms of potentially improved safety and reduction in medicolegal fees, the initial investment required remains substantial at present time. Furthermore, current evidence is mixed in this context. Machine learning and robotic assistance do not necessarily increase procedural efficiency, and the evidence for reducing learning curves is varied across surgical contexts [80]. Therefore, more evidence is needed to support the use of AI in RA across a wide range of techniques and trainee populations, in addition to a robust validation tool to evaluate these novel simulation systems.

## 8. Evaluation of Novel Simulation Systems

It is now apparent that SBT has become essential for the adoption of new technologies in RA. Nonetheless, a well-established validation tool for the evaluation of simulation training is crucial to ensure that the limited resources at our disposal are optimally utilised. One such validation tool is the “Kirkpatrick Model”, a widely used, four-level training evaluation method that benefits both learners and educators by elucidating the value and impact a specific training has had on a team.

It was in 1959 when Donald Kirkpatrick first published his ideas about training evaluation [81]; then, in 1975, he further defined them in his book *Evaluating Training Programmes*, which received widespread attention and adoption of his methodology. In 2016 James Kirkpatrick further refined and updated the methodology in a revised edition titled “*Kirkpatrick’s Four Levels of Training Evaluation*” [82]. Since then, the Kirkpatrick model has become an invaluable tool for the learning and development community to evaluate new models of training.

**Figure 1:**
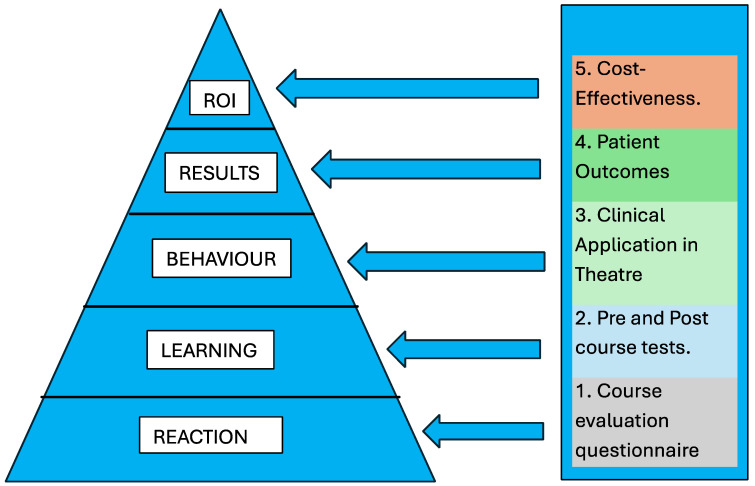
The Kirkpatrick Model as modified by Phillips.

The four levels of Kirkpatrick’s Evaluation Model (Figure 1) are:

1. **Reaction**: this involves the trainees and measures the extent to which they find the training agreeable, relevant and engaging. Their satisfaction levels are usually assessed using a feedback form, often referred to as a ‘Happy Sheet’. The biggest advantage of this level of assessment is that it is quick, simple, cheap and easy to conduct.
2. **Learning**: this level gauges the gain in knowledge and capability experienced by the trainee by conducting and comparing pre- and post-learning assessments. These assessments can be conducted via exams or an interview-style evaluation. Like the previous level, this level is relatively easy to set up and is useful for assessing quantifiable skills.
3. **Behaviour**: this level assesses the trainee’s application of the acquired knowledge and skills in a working environment. Compared to Levels 1 and 2, Level 3 requires a greater level of participation from trainees as well as prolonged observation by assessors to identify any changes in behaviour and whether the change is relevant and sustained.
4. **Results**: this final level of the original model measures the direct and overall impact of the trainee’s performance on the business or working environment. This involves individual assessments against agreed goals and is the toughest of the 4 levels.

An additional level, Level 5, **Return of Investment (ROI)**, was added by Jack Phillips in 2003 [83], which guides learning and development practitioners on how to calculate the ROI of training using data gathered from Kirkpatrick’s Level 4 Evaluation in a more actionable format. It is a measure of the cost-effectiveness of the overall training.

## 9. Conclusions and Future Directions

With the innovations in simulation-based training, we currently bear witness to nothing short of another “industrial revolution” of sorts. Various branches of the sciences are amalgamating with digital technology to transform the field of medicine for the betterment of mankind. This transformation has already revolutionised the journey of medical trainees from the classroom to bedside patient care. Its implications for the advancement of education and training in regional anaesthesia are immense and may ultimately fulfil its promise of improving perioperative patient experiences and pain management.

Emerging evidence strongly suggests that AI platforms can be incorporated into tools not only for simulation training on cadavers, VR or AR models but also in actual performance of nerve blocks on patients to improve block outcomes as well as minimise injury and harm.

There exists concern that the skills acquired from SBT may not transfer to clinical practice; hence, further large-scale studies are vital to achieving ascension to level 5 of the Kirkpatrick Model (cost-effectiveness).

The need of the hour is wider acceptance, more multicentric trials and validation of AI training models to overcome the steep learning curve during RA training. We also need to recognise that technology is a double-edged sword for which we in the practice of medicine have been witness to time and again, hence the urgent need for a universal AI governance framework, akin to an ethics committee, to guide enthusiasts in the direction of “*primum non nocere*”, our time-tested guiding principle.

We end this review with some pebbles of wisdom from Marhofer et al. [16]; “*There needs to be a balance between the evangelical fervour of the innovative enthusiast and the resistance of the clinical Luddite. Open minds, healthy scepticism, and a desire to work together are likely to benefit patients the most*”.

## Data Availability

Not applicable.
